# Investigating the effectiveness of a smart mental health intervention (inMind) for stress reduction during pharmacological treatment for mild to moderate major depressive disorders: Study protocol for a randomized control trial

**DOI:** 10.3389/fpsyt.2023.1034246

**Published:** 2023-03-14

**Authors:** Junhyung Kim, Cheolmin Shin, Kyu-Man Han, Moon-Soo Lee, Hyun-Ghang Jeong, Chi-Un Pae, Ashwin A. Patkar, Prakash M. Masand, Changsu Han

**Affiliations:** ^1^Department of Psychiatry, Korea University Guro Hospital, Korea University College of Medicine, Seoul, Republic of Korea; ^2^Department of Psychiatry, Korea University Ansan Hospital, Korea University College of Medicine, Seoul, Republic of Korea; ^3^Department of Psychiatry, Korea University Anam Hospital, Korea University College of Medicine, Seoul, Republic of Korea; ^4^Department of Life Sciences, Korea University, Seoul, Republic of Korea; ^5^Department of Psychiatry, College of Medicine, The Catholic University of Korea, Seoul, Republic of Korea; ^6^Cell Death Disease Research Center, College of Medicine, The Catholic University of Korea, Seoul, Republic of Korea; ^7^Department of Advance Psychiatry, Rush University Medical Center, Raleigh, NC, United States; ^8^Global Medical Education, New York, NY, United States

**Keywords:** stress, major depressive disorders, mobile health, mindfulness-based intervention, cognitive behavior therapy, relaxation therapy, cost-effectiveness, randomized controlled trial

## Abstract

**Background:**

Although psychological interventions for stress relief, such as cognitive behavioral therapy (CBT) and mindfulness-based stress reduction (MBSR), have been developed, they have not been widely used in treating depression. The use of mobile devices can increase the possibility of actual use by integrating interventions and reducing the difficulty and cost burden of treatment application. This study aims to determine whether “inMind,” an integrated mobile application for stress reduction, developed for the general population, decreases stress for patients with mild to moderate major depressive disorder during the pharmacological treatment period.

**Methods:**

This study is a single-blind, multicenter, randomized, controlled crossover trial. The App, developed in Republic of Korea, provides integrated interventions for stress reduction for the general population through three modules based on mindfulness-based stress reduction, cognitive behavior therapy, and relaxation sounds that are known to be effective in stress reduction (“meditation,” “cognitive approach,” and “relaxation sounds,” respectively). Participants (*n* = 215) recruited *via* medical practitioner referral will be randomized to an App first group (fAPP) or a wait list crossover group (dAPP). The study will be conducted over 8 weeks; the fAPP group will use the App for the first 4 weeks and the dAPP group for the next 4 weeks. During all study periods, participants will receive their usual pharmacological treatment. The Depression Anxiety Stress Scale-21 is the primary outcome measure. The analysis will employ repeated measurements using a mixed-model approach.

**Discussion:**

The App can potentially be an important addition to depression treatment because of its applicability and the comprehensive nature of the interventions that covers diverse stress-relieving models.

**Clinical trial registration:**

https://clinicaltrials.gov/ct2/show/NCT05312203, identifier 2021GR0585.

## 1. Introduction

Stress is a factor that causes depression as well as is caused by depression (1, 2). Stress is considered an important factor in both directions in depression because it affects suicidal behavior (3), social relationships (4) and depression itself (5). However, the chronic stress response in depression has not received much attention compared to symptoms such as depression and anxiety (6). Since the effect of antidepressant drug treatment requires several weeks of follow-up (7), an effective tool that can alleviate chronic stress in patients with depression during the treatment period is essential.

The transactional theory of stress explains stress as an interaction between a person and their environment (8, 9), suggesting that a person can deal with stress by identifying stress-related appraisals and modifying their cognition and coping (10). Mindfulness-based stress reduction (MBSR) and cognitive behavioral therapy (CBT) are two psychological approaches that could facilitate increased awareness of stress-related (and other) appraisals, re-evaluation or non-judgmental acceptance of these appraisals, and increased awareness of available options for responding appropriately.

The effectiveness of MBSR in depression among adolescents, young adults, and older adults has been suggested in previous studies (5, 11). These approaches pay less attention to thought-content reframing and behavior modification and more on modifying one’s connection between thoughts and behaviors through meditation practice (12). Mindfulness is characterized by non-judgmental awareness and acceptance of here and now experiences (including thoughts, feelings, sensations), and greater awareness of suitable behavioral choices available (13).

Among the variety of accessible psychiatric interventions, CBT has held a dominant position (14). It is one of the most widely utilized interventions and is recognized as a treatment of choice for depression, either alone or in combination with pharmacotherapy (15). The efficacy of CBT in reducing stress has also been shown in a previous meta-review study (16). CBT for stress-management consists of recognizing how thoughts (including appraisals), feelings, behaviors, and physical sensations interact to cause stress, and then utilizing that understanding to find strategies for preventing or modifying stress (17). Based on the CBT approach (18), possible strategies are recognizing and re-evaluating the accuracy of stress-related beliefs (appraisals), discovering stress-inducing behaviors and substituting more helpful behaviors, and identifying techniques to lower physiological arousal associated with stress (19–21). Through this process, the stress-related maintenance cycle of thoughts, feelings, behaviors, and bodily sensations can be disrupted and replaced with a stress-relieving maintenance cycle.

Stress occurs with a stress system response, involving the amygdala, hypothalamus, autonomic nervous system (ANS), and various hormone glands and organs (22). Several studies have found that chronic stress disrupts the balance of the ANS and causes hormonal system imbalance and inflammation represented by increased cortisol secretion, that affects the occurrence and exacerbation of depression (23). Symptomatic interventions that can reduce these bodily responses have been attempted in non-clinical settings. Listening to relaxing sounds has been shown to lower cortisol levels and help recover from stress (24). Moreover, Alvarsson et al. found that exposure to colors and sounds in the natural environment helps people recover quickly from stress (25).

Applying interventions for stress reduction in clinical settings for patients with depression can be challenging. MBSR and CBT for stress reduction are group or individual-based treatments conducted for 1–2 h per week for several weeks, thus, there is a barrier to their wider implementation due to significant time and economic costs (5, 11). In addition, the use of white noise is relatively advantageous in terms of time and economic cost; however, there is no way to provide it systematically. The recent decline in access to healthcare due to the COVID-19 pandemic has made these limitations even more pronounced (26).

Smart Mental Health Interventions (SMHI) are tools that help overcome these limitations. SMHI delivered *via* applications or text messaging have shown promise in addressing these concerns. They lower hurdles to accessing conventional mental health services, such as cost, stigma, and accessibility (27, 28). The digital nature of SMHI makes it possible to include various treatment components in one application, as well as to keep accurate records of users’ activities, that form the basis for providing appropriate interventions for individuals (29). In addition, current techniques enable bio-signals acquisition using mobile devices, that can help an individual take appropriate action by providing an objective measure of their stress (30, 31). While SMHI may vary in terms of strategy, focus, and mode of delivery, they appear to be as effective as conventional in-person treatment interventions (32).

Since several previous SMHI have tried to investigate the effects of transplanting MBCT, ACT, and CBT, provided by clinics, to mobile devices on psychiatric symptoms such as depression and anxiety (19, 32–36), most of them required direct participation of clinicians and patients. Therefore, to reduce the time cost, it would be helpful to develop SMHI with fully contactless intervention from the therapist. Considering the importance of stress management in the treatment process for depression, developing the adjunct role of SMHI that can be used for a short period for stress relief in depression treatment strategies is essential. However, to our knowledge, there has been no attempt to use SMHI for chronic stress management during depression treatment.

Other than psychiatric disorders, there are examples of SMHI use concerning work-related stress. For example, self-guided internet-based stress management intervention for workers, and mobile apps in connection to the COVID-19 pandemic that emphasized coping strategy development and resilience enhancement, demonstrated a moderate-to-large impact size for stress reduction (37, 38). However, in the context of depression’s susceptibility to stress and pathological cognitive structure, it would be beneficial to incorporate a broader range of interventions and an objective assessment of stress. Given the current heterogeneity and room for improvement in solutions related to stress reduction in patients with depression, there is a need for a well-designed intervention that integrates existing interventions, allowing participants to utilize various components freely and decrease costs by reducing direct intervention from therapists and time of use.

For this purpose, the “inMind” App (from hereon, the App), an SMHI tool that combines relaxing sounds, MBSR, and CBT modules, that can be used independently by users and is being used as a digital wellness tool for the general population in Republic of Korea^1^ is employed. The current study [Alleviating Stress by Mobile Application for Depression (ASMA-D)] investigates whether the App may be an effective solution to overcome the challenges mentioned above.

The aim of the ASMA-D study is to evaluate the efficacy of the App in reducing stress in patients with depression during treatment. We also aim to investigate the feasibility and acceptability of the 4-week mobile application intervention in this setting.

The study’s primary hypothesis is that applying this novel mobile application intervention for patients with mild to moderate major depressive disorder (MDD), for 4 weeks with usual care, will result in greater reduction of stress compared to usual care alone.

## 2. Materials and methods

### 2.1. Design

**FIGURE 1:**
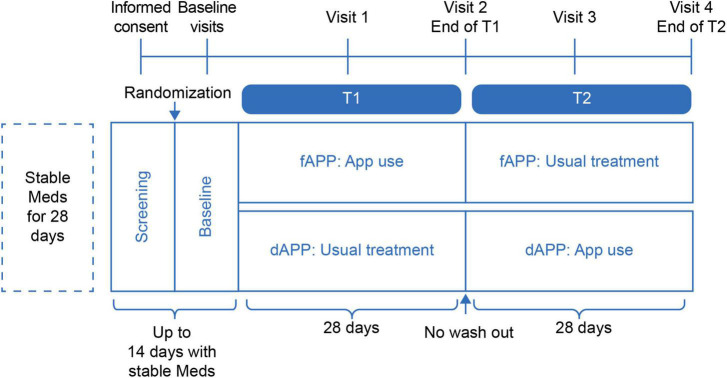
ASMA-D study schematic diagram.

**FIGURE 2:**
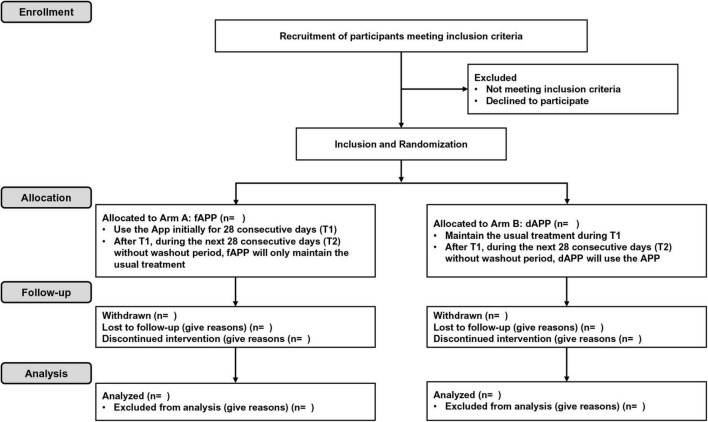
SPIRIT flow diagram of the ASMA-D trial.

This study is a single blind, multicenter, randomized, controlled crossover trial (Figure 1). All subjects who meet the inclusion and exclusion criteria will be randomly assigned to the App first group (fAPP) or the wait list crossover group (dAPP). Due to the intervention’s nature, this will be a single-blind study in which the results will be assessed blindly but participants will be aware of their group assignment. Participants assigned to fAPP group will use the App initially for 28 consecutive days (T1). During T1, participants assigned to the dAPP group will maintain their usual treatment. After T1, for the next 28 consecutive days (T2), dAPP will use the App and fAPP will maintain the usual treatment, without a wash-out period. We assume that there would be no carry-over effect as the App utilized in this study is not a curative treatment but rather a tool for relieving stress. Therefore, we have decided to use a cross-over design without a wash-out period. Recommendation for Interventional Trials (SPIRIT) flow diagram is presented in Figure 2.

### 2.2. Eligibility criteria

The inclusion criteria are as follows: (1) Adults between the ages of 19 and 65; (2) Those diagnosed with mild to moderate MDD in an expert interview evaluation according to the DSM-V diagnostic criteria [Score of 7–24 on the Hamilton Rating Scale for Depression (HAM-D)]; (3) Those on stable medication for 28 days prior to study participation; and (4) Those who provide informed consent and voluntary participation.

The exclusion criteria are as follows: (1) Difficulty using smart phones or inability to independently use the App; (2) A diagnosis of severe MDD in an expert interview evaluation according to the DSM-5 diagnostic criteria (score of 25 or more on HAM-D); (3) Severe mental disorders (current or in the past), including MDD with psychotic features, bipolar affective disorder, personality disorder, obsessive compulsive disorder, autism spectrum disorder, and substance use disorder; (4) History of brain injury, epileptic seizures, intellectual disability, or cognitive disorders; (5) History of severe physical disorders, including cancer, tuberculosis, severe cardiovascular disease, etc.; and (6) Individuals participating in other cognitive behavioral therapies or activities related to stress relief.

### 2.3. Generic description of the App

**FIGURE 3:**
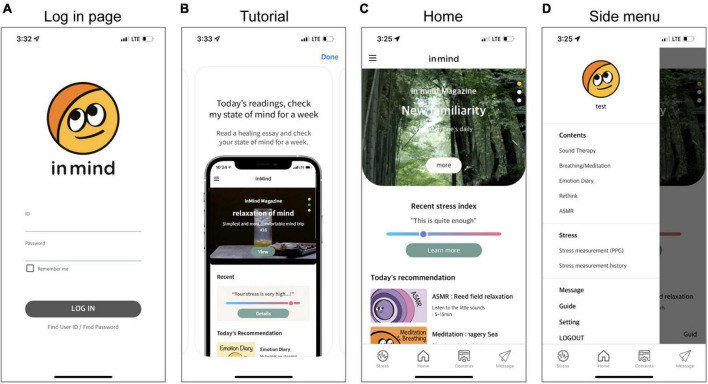
Screenshots of the App user interface. All participants log in with their ID and password on the log-in page **(A)**. Participants can watch a tutorial about application use at any time **(B)**. The Home screen recommends treatment programs based on the results of participants’ recent objective stress monitoring, and participants can access the desired content from the menu bar **(C)**. Participants can also directly access content by category through the side menu **(D)**.

The App implements mobile-based strategies for stress relief that can be used on most Android and iOS-based mobile devices. The stress relief strategies involve three modules: meditation, the cognitive approach, and relaxation sounds. Additionally, the App includes a module for objective stress monitoring. The App was originally designed and developed in Korean, but English, Chinese, and Japanese versions are also available. For use in the treatment environment, the manufacturer also provides a service that allows therapists to check their patients’ application usage and their stress level measured by the stress monitoring module. The general user interface of the App is shown in the screenshot in Figure 3. The specific user interface and contents for each module are described below.

#### 2.3.1. Meditation module

**FIGURE 4:**
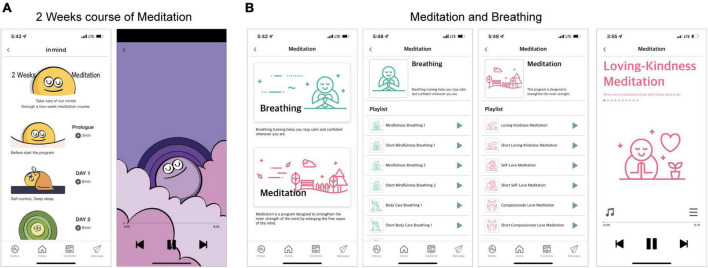
Screenshots of the meditation module. This module has two parts (**A**: “Two weeks course of meditation” and **B**: “Meditation and breathing”), wherein participants select one of several choices presented as a list and listen to the guided audio recording. The content was presented in Korean, but the examples in this figure are presented in English to aid understanding for international readers.

The meditation module in the App is modified to be mobile and self-utilizing based on the MBSR program created by Jon Kabat-Zinn et al. (37). The meditation module consists of two components: “two weeks course for meditation” and “meditation and breathing.” The “two weeks course for meditation” contains 14 topics related to MBSR, and provides 5–15 min with backgrounds and voices that can help stabilize the mind and focus (Table 1 and Figure 4). It helps participants gain a better understanding of MBSR and learn how to train in “meditation and breathing.” There are nine topics (four training topics centered on breathing methods and five topics centered on meditation) in the “meditation and breathing” component, provided in both long (15 min or longer) and short (less than 10 min) forms, with backgrounds and voices that can help stabilize the mind and focus (Table 1 and Figure 4). Therefore, participants can utilize the content from relevant topics according to their situation.

**TABLE 1:** Titles and contents of the meditation module.

Component	Title
Two weeks course for meditation	Prolog, before entering the meditation, introduction
	DAY 1- Self-regulation
	DAY 2- Feel the nature through breathing
	DAY 3- Come back to your breath at any time
	DAY 4- Relaxation by focusing on the object
	DAY 5- Image training, the power of imagination
	DAY 6- Come back to here and now through breathing
	DAY 7- Observation of thoughts and emotions, returning to the here and now
	DAY 8- Observation of the senses, returning to the here and now
	DAY 9- Mindfulness yoga, Follow your mind through movement
	DAY 10- Awakening nature through eating
	DAY 11- Walking meditation, meditating while walking
	DAY 12- Loving kindness meditation, warm mindfulness
	In closing, what kind of meditation is appropriate for me?
Meditation and breathing	Meditation 1–Loving-kindness meditation, getting closer to yourself through the change of feelings you feel while thinking about charity
	Meditation 2–Self-compassion meditation, recognizing the connection between you and the world and being kind to yourself
	Meditation 3–Compassion meditation, cultivate one’s own mind through supporting compassionate objects
	Meditation 4–Guided image method- Forest, imagining walking in the forest to relax and recharge the mind
	Meditation 5–Guided image method- Sea, imaging relaxation and recharging the mind through imagining walking on the sea
	Breathing 1–Mindful breathing method 1, the sensations felt in my body and the changes that occur in my body through breathing
	Breathing 2–Mindful breathing method 2, through breathing, the sensations felt in my body and body tension are relieved and my mind is relaxed
	Breathing 3–Body-awareness breathing method 1, relaxation through awareness of my whole-body senses
	Breathing 4–Body-awareness breathing method 1, improving attention through awareness of my whole-body senses

#### 2.3.2. Cognitive approach module

**FIGURE 5:**
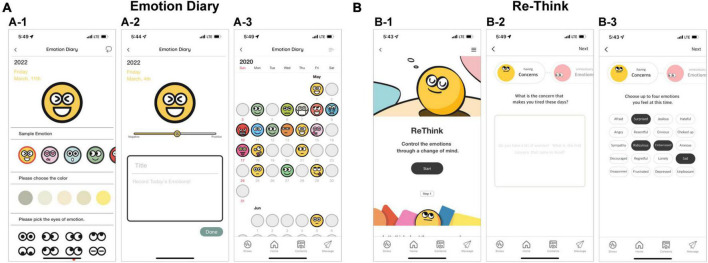
Screenshots of the cognitive approach module. The module includes two parts (**A**: “Emoji and Emotion Diary” and **B**: “Re-Think”). Emoji is a tool for expressing varied emotions; the participant can choose what is presented as a sample or create their own Emoji by selecting a color or shape such as eyes and mouth (A-1). The diary is created by checking the emotional intensity related to the previously created Emoji through the slider and entering the title and details (A-2). Participants can see the content of their diary and the emoji of any particular day through the calendar (A-3). In all steps in the “Re-Think” part, participants write answers to the questions displayed on the screen (B-2), and select emotion cards (B-3). The content was presented in Korean, but the examples in this figure are presented in English to aid understanding for international readers.

The cognitive approach module consists of two components, “emotional diary” and “ee-Think.” In “emotional diary,” participants can express their feelings through emojis and journaling (Figure 5A). The transactional theory of stress suggests that keeping a diary can help manage stress through re-formulating one’s own situation-related stress experience (39, 40). Therefore, the “emotional diary” component can help participants manage their stress through keeping an emotional diary.

The “re-think” component aims to help participants practice changing their negative thoughts into positive ones by highlighting the inter-relationships between thoughts, feelings, physical sensations, and behaviors and dealing with biased thinking that induces negative emotions (19–21). The “re-think” component consists of three steps (Figure 5B). First, participants express their concerns that cause them stress, in detail. Second, they compare the expressed emotions and concerns, and then try to see the actual situation rather than the related emotions. Third, participants advise themselves from a third-person perspective based on the contents expressed up to the previous point. Then, participants are guided to think about the change of emotions about their concerns from the starting point through the previous steps and put it in writing.

#### 2.3.3. Relaxation sound module

**FIGURE 6:**
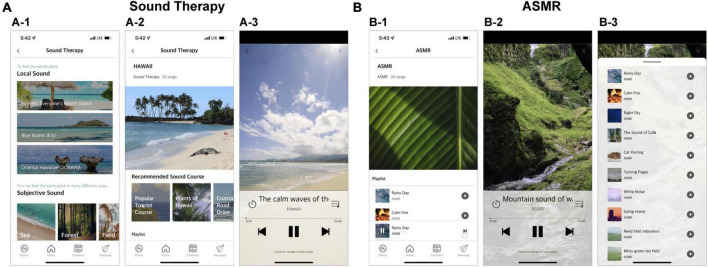
Screenshots of the relaxation sound module. The module includes two types of sounds (**A**: “Sound Therapy” and **B**: “ASMR”). Participants can select a region of natural scenery they would like to hear and chose from the curated “Sound therapy” list (A-1). Various types of videos are prepared for each region, and participants can select from them (A-2). Panel (A-3) shows the video playback screen. Participants can click the video they want from the list presented in the ASMR component (B-1). Panel (B-2) shows the screen where the video is played in “ASMR.” Both “Sound Therapy” and “ASMR” can be accessed as a list from the playback screen (B-3). The content was presented in Korean, but the examples in this figure are presented in English to aid understanding for international readers.

This component involves two types of sounds: the sounds found in the natural environment (Sound Therapy) and those associated with Autonomous Sensory Meridian Response (ASMR), known to help recover from stress (41). Since there is no consensus on which type and delivery of sound is effective, this category tries to provide a wide range of experiences to help participants choose the sound they prefer. To suit the various personal preferences, the manufacturer provides 117 sounds recorded in 3 places (Jeju Island, The Republic of Korea; Okinawa, Japan; and Hawaii, USA) in the Sound Therapy component and 30 sounds in ASMR. Figure 6 presents a screenshot of the user interface of this module, with each component.

#### 2.3.4. Objective stress monitoring

**FIGURE 7:**
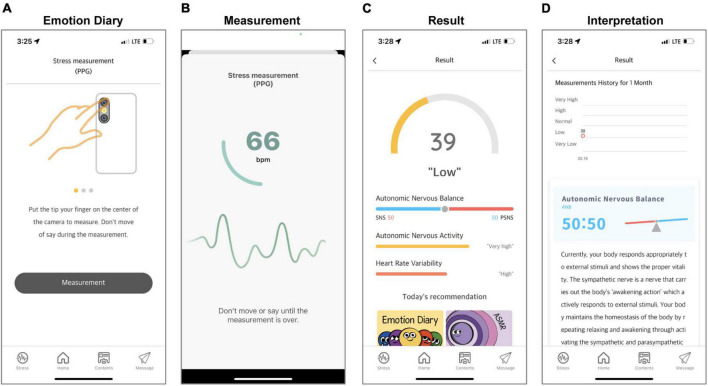
Screenshots of the objective stress monitoring. The “Guide” shows how to measure PPG and guides participants on situations to pay attention to during PPG measurement **(A)**. When the measurement starts, the measured pulse rate is displayed on the screen, and the progress is displayed in a circle to check that 1 min has elapsed **(B)**. When the measurement is completed, autonomic nervous balance, autonomic nervous activity, heart rate variability, and stress index are displayed based on the results **(C)**. Participants can see the change in their results on the result screen and check the interpretation of the results **(D)**. The content was presented in Korean, but the examples in this figure are presented in English to aid understanding for international readers.

For the objective measurement of stress, heart rate variability (HRV), that reflects stress response *via* ANS activity, can be measured by acquiring the photoplethysmography (PPG) through a mobile camera (42). To record the pulsatile PPG signal and calculate intervals between R-peak (RR intervals) intervals (R-peak interval), the module built into the App was designed for each phone based on the algorithm of a previous study (43), that showed relative high performance (relative errors < 5%, Pearson correlation > 0.9, and standard estimated error < 5 beats-per-minutes) with reference to a medical device (Beurer BC 08, Beurer GmbH, Germany). The App instructs all participants not to speak and make any movement during a 1-min recording with visual guidance (Figure 7).

Continuous HR signals are normalized to unit variance and then power spectra were determined using the Welch periodogram method (44). Total power in the frequency range (TP: 0–0.40 Hz) is divided into low frequency (LF: 0.04–0.15 Hz) and high frequency (HF: 0.15–0.40 Hz) (45). The TP, LF, and HF components are calculated in normalized units. TP is interpreted as autonomic nervous activity, and LF/HF reflects autonomic nervous balance. In addition, HRV is calculated by obtaining a histogram for each heartbeat time interval and dividing the total frequency by the maximum of the density distribution (46). The results show autonomic nervous activity, autonomic nervous balance, and HRV (Figure 7). To help participants interpret their results, the manufacturer has developed a stress index that converts the sympathetic nerve activation level into a score between 0 and 100, with a higher score indicating higher stress.

### 2.4. Setting

Three university hospitals in Republic of Korea participating in the study will recruit patients with depression to use the App. The Korea University Guro Hospital and Korea University Anam Hospital are in the inner center of the Capital, and Korea University Ansan Hospital is in Ansan city, on the outskirts of the capital area. Each hospital is a general hospital, and the Korea University Guro Hospital will lead the ASMA-D study. For patient recruitment, 96 patients (48 interventions and 48 controls) in the Korea University Guro Hospital, and 60 patients (30 interventions and 30 controls) each from Korea University Anam Hospital and Korea University Ansan Hospital are expected.

Candidates participating in the current study can use the “inMind” application free of charge. During the 8-week study period, participants will be provided with transportation expenses worth KRW 10,000 for every 2-week hospital visit. In addition, participants who complete 22 days or more out of 24 days (excluding holidays) according to the planned schedule without interruption or dropout during the 4-week inMind usage period will receive a monetary reward worth KRW 100,000.

### 2.5. Recruitment process and screening for enrollment

Patients with depression who visit the hospital and are interested in the advertisements posted in the participating hospitals will be screened. Once interested patients contact the research team, they will be screened for eligibility by the psychiatrist according to the above-described criteria. After eligibility is established, information regarding the study will be given to the prospective participants. Once the patient agrees to participate, informed consent will be acquired, and randomization will occur only after the participant is enrolled.

### 2.6. Randomization

Eligible volunteers will be randomly assigned to the fAPP or dAPP groups. Randomization is conducted using block randomization by each clinical center. To prevent bias toward one treatment group, eight patients will be configured in blocks and will be randomly assigned to the fAPP and dAPP groups within each block. For consistent allocation, randomization will be done using a pre-built program. This program is created by Korea University Guro Hospital to blind the researcher from assignment information in advance. The allocation sequence was prepared by researchers who did not participate in this protocol, and was used in the pre-built program. Researchers at each institution can conceal the sequence through the issued ID and password, and the process remains in a log.

### 2.7. Interventions

#### 2.7.1. Administration of the App

Participants will be instructed to use the App autonomously for 4 weeks at home, in addition to their usual treatment, during the period of using the App (T1 for fAPP group, T2 for dAPP group). According to their assigned group, each participant will receive basic instructions from the researcher on how to use the App.

As the purpose of using the App is to obtain an additional stress relief effect in the usual treatment for patients with depression, the schedule for the App will not be strictly controlled; the researchers will provide a 4-week schedule as a guideline to ensure the use of more than the minimal. As a minimum standard for usage, the schedule will guide participants to use the components of each module at least once a day, 6 days a week or more, and the use of additional content is left to the participant’s discretion. However, regarding the meditation module, the participants will be guided to conduct training after completing psychoeducation in consideration of the characteristics of the content. The App has implemented a system that alerts the user twice per day at a time they select if it is not utilized at least once per day. The researcher will help participants who have provided permission to activate the alarm system in the App. However, whether a participant follows these guidelines is not a determining factor in excluding respective participants’ data.

#### 2.7.2. Usual treatment for depression

All participating patients will continue all of their usual treatments for depression during the research period. To minimize the confounding effect of medication, the use of new psychiatric medications, including antidepressants and benzodiazepines, will be limited. The use of zolpidem and zopiclone for insomnia is possible. To participate in the study, a stable dosing period of 4 weeks is required, so considering the course of psychiatric drug treatment, in general, the possibility of changing medication will not be high. However, psychiatrists will address any changes in depression symptoms of participants immediately after they are identified. Should there be medication changes, participants’ will be entitled to withdraw from the study, and our research team will assess their situation to see if they need another treatment.

### 2.8. Assessments and outcomes

Assessments will be obtained through the clinician rating scale and self-rating scales during hospital visits. Table 2 presents the schedule for assessments. Assessments conducted at the hospital visits were acquired at the baseline (V_0_) and every 2 weeks (V_1_, V_2_, V3, and V4) during T1 and T2. The baseline assessments will be collected when participants are enrolled and will include their sociodemographic and clinical characteristics. All instruments have been translated to Korean with reliable psychometric properties. The data collection process implanted in the objective monitoring module of the mobile App will be activated through an ID issued after enrollment and randomization.

**TABLE 2:** Schedule for assessments.

Assessments	Baseline	Visit			
		**V1**	**V2**	**V3**	**V4**
Mini international neuropsychiatric interview	V				
Depression, anxiety, and tress scale	V	V	V	V	V
Perceived stress scale	V		V		V
Posttraumatic embitterment disorder scale	V		V		V
Nine-item patient health questionnaire	V	V	V	V	V
Hamilton depression rating scale	V	V	V	V	V
Intervention engagement					V

### 2.9. Diagnostic interview

The Mini International Neuropsychiatric Interview (MINI) is a brief, organized diagnostic interview used to make psychiatric diagnoses using the DSM-IV and ICD-10 codes. The MINI was developed as a brief yet reliable structured psychiatric interview for multicentric clinical trials and epidemiology investigations (47).

### 2.10. Primary outcome measures

#### 2.10.1. Depression, anxiety, and stress scale (DASS-21)

The DASS-21 is s a self-report measure of anxiety, depression, and stress, developed by Lovibond for use in varied situations (48). Eun-Hyun Lee developed the Korean version of DASS-21 in 2018 (49). This version was evaluated in Korean-speaking samples and demonstrated that the items had been translated and adapted adequately and appropriately. The Korean version of DASS-21 was assessed on 481 Korean adults, and the Cronbach’s α was 0.93.

### 2.11. Secondary outcome measures

#### 2.11.1. Perceived stress scale (PSS)

The perceived stress scale (PSS) is designed to assess the respondents’ perception of their stress as unpredictable, uncontrollable, and overwhelming (50). The current study will utilize the validated Korean version of the PSS, which is a 10-item self-report questionnaire using a five-point Likert scale ranging from 0 (never) to 4 (very often). Total scores range from 0 to 40, with higher scores indicating increased perceived stress (Cronbach’s α = 0.819) (51).

#### 2.11.2. Posttraumatic embitterment disorders scale (PTED)

Posttraumatic Embitterment Disorder Self-Rating Scale (PTED Scale) was developed to evaluate the characteristics of embitterment reactions to negative life events (52). The PTED Scale, that consists of 19 items, asked participants to score their reactions to each negative life event that occurred in the recent years using a five-point scale ranging from 0 (not true at all) to 4 (extremely true). The PTED Scale was translated and validated in Korean with high internal consistency (Cronbach’s α = 0.962) (53).

#### 2.11.3. Nine-item patient health questionnaire (PHQ-9)

The Patient Health Questionnaire (PHQ-9) is a nine-item version of the historical Primary Care Evaluation of Mental Disorders (PRIME-MD), that was shortened to maximize clinical usefulness (54). The total score can range from 0 to 27, and each item is rated on a scale of 0–3. Mild, moderate, and severe depression severity levels are represented by the total scores of ≥ 5, ≥ 10, and ≥ 15, respectively (55). The PHQ-9 was translated and verified in Korean with strong internal consistency (Cronbach’s α = 0.86) (56).

#### 2.11.4. Hamilton depression rating scale (HAM-D)

The Hamilton Depression Rating Scale (HAM-D) is an inventory of questions used to detect and assess the severity of the signs and symptoms of depression in individuals who have been diagnosed with clinical depression (57). The 17-item version of the HAM-D is more widely used than the 21-item version, that includes four extra items that assess symptoms related to depression, such as paranoia and obsession, rather than the severity of the depressive symptoms. The HAM-D was translated and validated in Korean with good internal consistency (Cronbach’s α = 0.76) (58).

#### 2.11.5. Intervention engagement

Track and change functionalities (log files) will be used to evaluate activities on the app, such as the number of visits, duration between logins, and the number of usages of each category. Usage data on the App online server will also provide information on how patients utilize online modules (frequency, length, order, completion), how they rank them, and how they adhere to them. All patient data from the server will be secured using Hypertext Transfer Protocol Secure, ensuring that no third parties may see it while it is being transferred. Only the research assistants will be allowed to request and evaluate patient data since data access from the server is additionally authorized through a pass provided by the study coordinator.

### 2.12. Sample size and power calculations

To the best of our knowledge, there are no studies on stress-reduction programs for depression patients using a mobile application; hence, we have assumed the sample size based on the effect size of 0.28, being half of the effect size of 0.56 reported in a previous study that applied MBSR in patients with depression (59). For all patients with depression, the change in DASS-21 score from pre- to post-administration of the App would be analyzed using analysis of variance for a 2 × 2 crossover design (60). As we have assumed no carry-over effects, this was not considered in calculating the sample size. Sample size calculations were conducted using the statistical software G * Power 3.1.9.2 (61). The minimum sample was identified as 172 (fAPP = 86, dAPP = 86) based on an estimated between-group effect on post-intervention stress outcomes between the fAPP and dAPP with a power of 0.95 and an alpha of 0.05. Therefore, the current study will enroll 215 participants and allow for up to a 20% loss to follow-up, expecting a minimum effective sample size of 172 individuals.

### 2.13. Data analyses

Data analysis will adhere to CONSORT rules (62), i.e., primarily on an intention-to-treat basis. The pre-randomization scores in the two conditions prior to receiving the intervention will be compared to ensure absence of significant differences between them on the outcome measures. Then, a multilevel linear mixed model will be computed, successfully managing missing data. The first evaluation of participants with missing data will assume that data are missing at random in the linear mixed model. Multiple imputation, last observation carried forward imputation, and assessment of the per protocol completers will be used to assess the sensitivity of this assumption, which should minimize the number of missing data. All measurement items will be incorporated into the model. The parameters will be estimated using a restricted maximum likelihood approach. If baseline differences are found, they will be included as variables in the linear mixed model. All analyses will be performed with SPSS v26.0.

### 2.14. Data management and protection

Most data, including the usage data on the App, will be documented during visits by the research assistant at each participating hospital; the original forms will be stored in a closed cabinet. Additionally, all unidentifiable data will be saved in a Dropbox-based central data management system^2^ weekly. Identifying information will be kept only in a local database isolated from the external network with restricted access. Since the central data management system is mirrored, data confidentiality and accessibility are always guaranteed. The permission to access data will be managed by the study coordinator. We will conduct self-inspection on consent forms and case records through weekly research meetings.

If a patient withdraws their participation in part or full, it will be recorded in the records, and no additional data (as determined by the patient) will be added. The patient’s data will not be used in the analysis and, if desired, will be removed from the project database (only data already used in studies and project outcomes will be excluded). After the data collection is complete, all identifying variables will be physically removed from the rest of the data. The analysis will be carried out in a purely pseudonymous way.

### 2.15. Ethical approval

All procedures conducted during this trial with human participants will be carried out in compliance with institutional ethical standards. All research procedures have been approved by an Institutional Review Board (IRB) at each participating site: Korea University Guro Hospital (IRB Reference No. 2021GR0585), Korea University Anam Hospital (IRB Reference No.2021AN0596), and Korea University Anam Hospital (IRB Reference No. 2021AS0391). This protocol has been prepared in accordance with the SPIRIT 2013 statement (63). The study was registered under ClinicalTrials.gov 2021GR0585 on 31 March 2022 and will be conducted following the CONSORT guidelines (62).

Participation will be voluntary and based on informed consent. All eligible participants will be given oral and written information about the study. Specifically, they will be informed that they may leave the study at any time, without providing any explanation, and that this decision will in no way affect their regular treatment at the center. The treatments will be carried out by qualified and expert professionals. In addition, according to the existing knowledge in this field, we have not found any potential risks for the participants; however, the appearance of any important clinical change involving any type of risk would not only imply removal of the participant from the project, but also their referral for specialized study and care.

## 3. Discussion

As a mobile application for stress relief, the App is a potentially important addition to depression treatment because of its applicability and comprehensive nature of the interventions that covers diverse contents for stress relief. It employs an integration of traditional mindfulness meditation including cognitive behavioral techniques, in addition to the objective monitoring of stress using HRV and relaxation sounds. This study aims to investigate the effectiveness of the App as an adjunctive intervention in patients with depression. It will explore if participants show improvement in stress after engaging with the App compared to usual treatment. The study will also explore the effectiveness of the App in improving anxiety and depression.

The study participants will be a subset of individuals with mild to moderate depression, whose mental health has considerable impact on their development, adversely affecting family wellbeing and community costs in terms of lost workdays and costs associated with medical treatments. The availability of treatment options for stress reduction during the treatment for depression is frequently limited through insufficient therapists, geographical remoteness, long waiting times, and high costs. In this context, an App could easily be delivered to patients with depression without having a long waiting period. As it provides not only meditation, but also a wide range of relaxation sounds, it can provide options that can be used according to the individualized needs and characteristics of patients. The short duration of the intervention can enable the program to be cost-efficient.

## Ethics statement

The studies involving human participants were reviewed and approved by the Institutional Review Board (IRB) at each participating site: Korea University Guro Hospital (IRB Reference No. 2021GR0585), Korea University Anam Hospital (IRB Reference No. 2021AN0596), and Korea University Anam Hospital (IRB Reference No. 2021AS0391). The patients/participants provided their written informed consent to participate in this study.

## Author contributions

CH was the chief investigator. JK was the principal investigator. CS and K-MH were co-supervisors. CH had oversight of the project design which was developed jointly with JK with advice from M-SL, H-GJ, C-UP, AP, and PM. AP and PM provided statistical advice for the design of the data collection and analysis and randomization. M-SL, H-GJ, and C-UP provided overall advice and support for the project. All authors read, amended, and approved the manuscript.

## Footnotes

1
http://www.demand.co.kr

2
https://dropbox.com
